# Anthracycline Cardiotoxicity in a Patient with Diffuse Large B-Cell Lymphoma: A Case Report

**DOI:** 10.7759/cureus.11038

**Published:** 2020-10-19

**Authors:** Francisco Teixeira da Silva, Rita Morais Passos, Alexandra Esteves, José Carvalho, Manuel Ferreira

**Affiliations:** 1 Internal Medicine, Unidade Local de Saúde do Alto Minho (ULSAM), Viana do Castelo, PRT; 2 Critical Care Medicine, Unidade Local de Saúde do Alto Minho (ULSAM), Viana do Castelo, PRT

**Keywords:** lymphoma, chemotherapy-related toxicity, cardiotoxicity, cardiomyopathy

## Abstract

Cardiotoxicity is a relevant side effect of cancer therapy that leads to increased patient morbidity and mortality. It is fundamental to understand and remember it as a cause of disease since accurate and timely diagnosis is crucial. We present the case of a patient who developed chemotherapy-associated cardiac dysfunction after receiving treatment for a diffuse large B-cell lymphoma. Clinical history, echocardiography, and differential diagnosis led us to attribute her condition to heart failure (HF) due to early doxorubicin-induced cardiomyopathy. The patient outcome was favorable. We discuss the pathogenesis and incidence of anthracycline-induced cardiotoxicity as well as strategies for its detection, prevention, and treatment.

## Introduction

Several drugs used as anticancer agents are associated with cardiotoxicity, but the most frequent are in the anthracycline group. Clinical presentation may range from immediate or acute toxicity (such as fatal arrhythmias or myocarditis) to heart failure (HF). Doxorubicin (Adriamycin) belongs to the anthracycline group and has been used in cancer treatment since the 1960s [1]. Its action produces oxidative stress, which seems to play a role in the cellular mechanisms that cause long term effects [2,3].

## Case presentation

A 79-year-old female patient with a previous medical history of hypertension, dyslipidemia, and atrial fibrillation was diagnosed with follicular non-Hodgkin lymphoma presenting with areas of fast-growing diffuse large B-cell lymphoma. She received therapy with R-CHOP protocol that includes rituximab, cyclophosphamide, hydroxydaunorubicin hydrochloride (doxorubicin hydrochloride), vincristine (Oncovin), and prednisone. Six treatment cycles were completed without reported adverse effects. A total cumulative dose of doxorubicin of 550 mg/m^2^ was administered; cumulative doses of rituximab, cyclophosphamide, vincristine, and prednisone were 7.7 g/m^2^, 8.4 g/m^2^, 12 mg/m^2^, and 3 g/m^2^, respectively. As per protocol, a transthoracic echocardiogram (TTE) was performed prior to initiating therapy; it showed a slightly increased left atrium, mild pulmonary hypertension, and a left ventricular ejection fraction (LVEF) of 73%.

Six months after completing treatment, she was admitted to our emergency department (ED) with exertional dyspnea, orthopnea, and decreased effort capacity. No previous history of chest pain or angina was noted. The symptoms had been present and were gradually worsening throughout the previous month.

**Figure 1:**
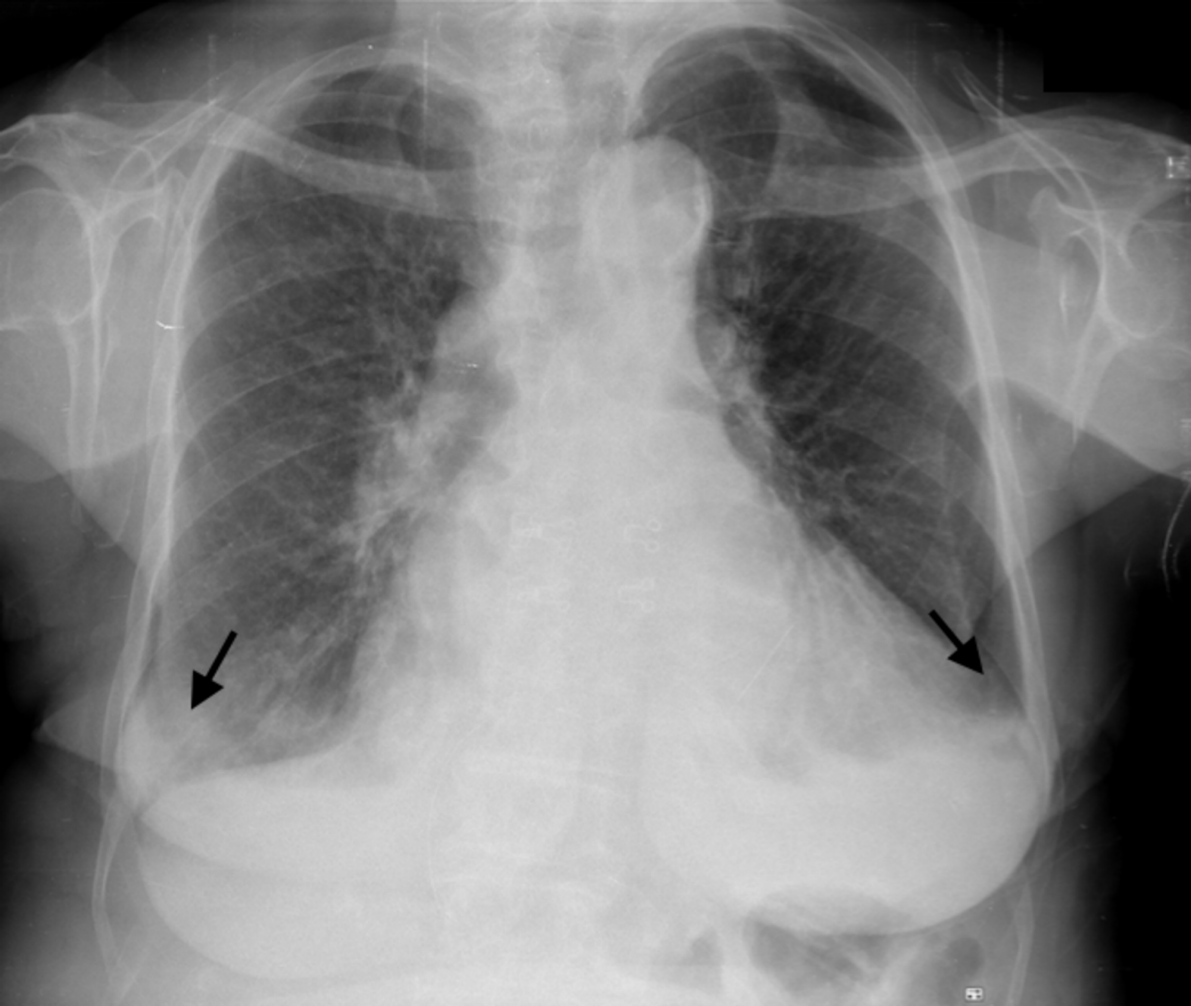
Chest radiograph Postero-anterior view X-ray shows a congestive pattern with blunt costophrenic angles (black arrows) suggesting bilateral pleural effusion.

Physical examination revealed bilateral pretibial edema, tachycardia, and hypotension but no venous jugular distension. On pulmonary auscultation, bilateral crackles were present. A chest radiograph showed a congestive pattern with bilateral pleural effusion (Figure 1). An electrocardiogram (EKG) showed atrial fibrillation with rapid ventricular response; cardiac biomarkers were slightly elevated with a high sensitivity troponin I of 102 pg/mL (normal range <15 pg/mL) and a brain-type natriuretic peptide (BNP) of 737.2 pg/mL (Table 1). Serial cardiac enzymes remained only marginally elevated and were not suggestive of myocardial infarction. 

**Table 1:** Laboratory workup Htc: hematocrit; MCV: mean cell volume; MCHC: mean corpuscular hemoglobin concentration; CRP: C-reactive protein; BNP: brain natriuretic peptide.

Tests	Reference values	Results
Haemoglobin (g/dL)	11.8 - 15.8	10.3
Htc (%)	36 - 46	33.4
MCV (fL)	80,4 - 96,4	88.1
MCHC (g/dL)	31,7 - 35,7	27.2
Leucocytes (μL)	4.0 - 10.0	4.23
Neutrophils (μL)	1800–7700	2200
Lymphocytes (μL)	800–4000	1400
Platelets (10^9^/uL)	150 - 400	203
Glucose (mg/dL)	70 - 110	90
Urea (mg/dL)	17 - 43	36
Creatinine (mg/dL)	0,6 - 1,0	0.82
Sodium (mmol/L)	136 - 145	140
Potassium (mmol/L)	3,5 - 5,1	3.4
CRP (mg/dL)	0-01-0.82	1.97
Total billirubin (mg/dL)	0.3 - 1.2 /	0.55
Alkaline phosphatase (UI/L)	30 - 120	162
Gamma-glutamyl Transferase (UI/L)	<55	78
Aspartate Transaminase (UI/L)	8 - 35	44
Alanine Transaminase (UI/L)	10 - 45	30
BNP (pg/mL)	<100	737.2
Mioglobin (ng/mL)	1 - 147	125
High sensitivity troponin I (pg/mL)	<15.6	102.5

She was classified as profile B (“wet-warm”) and a class III on the New York Heart Association (NYHA) Functional Classification for HF. She was admitted for heart rate control and diuretic treatment. A new TTE was performed and showed global hypokinesia but no segmental wall motion abnormalities, moderate to severe aortic and tricuspid dysfunction, moderate pulmonary hypertension, and a significant decrease in LVEF now at 45%. A diagnosis of early toxic cardiomyopathy secondary to doxorubicin was made.

Upon clinical stability, prognosis-modifying drugs were titrated to the maximum tolerated doses of beta-blocker, angiotensin-converting enzyme (ACE) inhibitor, and aldosterone receptor antagonist. A loop-diuretic was also prescribed for congestion management. Patient evolution was favorable as noted with progressive clinical improvement, increasing effort tolerance, remission of leg edema, and improvement to a class I of NYHA Functional Classification. After discharge, she remained asymptomatic. A TTE performed one year after diagnosis showed improved contractility and increased LVEF to 60% (remaining aspects were similar to those described previously).

## Discussion

Chemotherapy cardiotoxicity is characterized as type I or type II based on the agent's effect on cardiomyocytes. Type I cardiotoxicity is irreversible as it leads to cardiomyocyte death (via necrosis or apoptosis). Type II cardiotoxicity is caused by cardiomyocyte dysfunction and, as such, may be reversible [2]. The main mechanism of anthracycline cardiotoxicity is type I. It is thought to occur by inhibiting topoisomerase 2β and the resulting activation of cell death pathways and inhibition of mitochondrial biogenesis [4]. However, recent studies seem to suggest that cardiomyopathy is mostly reversible if detected early and treated promptly [5].

Regarding diagnosis, TTE is the preferred imaging modality, but equilibrium radionuclide angiography (ERNA), cardiac magnetic resonance imaging, and cardiac computed tomography can also be useful [2]. Increased BNP, its N‐terminal fragment (NT-pro-BNP), or troponin may and should be used to detect pre-symptomatic cardiotoxicity in patients treated with anthracyclines, either undergoing chemotherapy and after completing treatment [5].

Cardiotoxicity following anthracycline therapy is defined as a decline in LVEF of at least 5% to less than 55% with accompanying signs or symptoms of congestive HF, or a decline in LVEF >10% to below 55% without accompanying signs or symptoms [6]. It can present as acute or/and subacute effects: electrocardiographic changes, ventricular and supraventricular arrhythmias, cardiac conduction abnormalities (atrioventricular or branch block), ventricular dysfunction, myocarditis (which can account for up to 11% of cases [1]), and pericarditis have been described [7]. Chronic cardiomyopathy presenting as new-onset HF is the most common presentation; it can be defined as early if it begins within a year of ending chemotherapy, or late if it develops after that period [7].

There have been several risk factors associated with an increased risk of anthracycline-induced cardiotoxicity. These include age over 65 or under 18 years; female gender; hypertension; preexisting cardiac disease; concomitant use of mediastinal radiation therapy; coexisting treatment with cyclophosphamide, paclitaxel, or trastuzumab; cumulative anthracycline dose; and higher individual anthracycline doses [2,4]. The incidence of cardiac events escalates as cumulative doxorubicin doses increase and have been estimated at 32%, 54%, and 65% for doses of 400, 500, and 550 mg/m2, respectively [8]. Genetic factors to increased anthracycline cardiotoxicity susceptibility have also been identified: iron overload conditions such as hereditary hemochromatosis (with C282Y HFE gene mutation) are recognized as risk factors [4].

It is important to keep in mind that anthracycline-induced cardiotoxicity can be detected at a preclinical phase; early treatment interventions can change disease course [5]. That is why prevention is central for managing anthracycline toxicity. Possible strategies include prolonged infusion time, dose-sparing regimens or suspension of the drug, liposomal formulations, and concomitant administration of dexrazoxane (an iron chelator that has as a documented cardioprotective effect) [7,9]. Development of screening programs, including routine TTE, assessment of troponin values during chemotherapy, and a multidisciplinary team (including cardiologists, hematologists, oncologists, and nurse practitioners) are recommended for the management of these patients [5,7,9].

There is no specific treatment for anthracycline-induced HF. Standard therapies for congestive HF (either with preserved or reduced ejection fraction) should be employed, including ACE inhibitors, beta-blockers, and loop diuretics for volume overload management [2,4,5].

In our patient, absence of chest pain, borderline and stable myocardial necrosis biomarkers, timing and exposure to a cardiotoxic drug, and no segmental wall motion abnormalities on TTE advocate for a diagnosis of a non-atherosclerotic cardiomyopathy diagnosis and are not suggestive of myocardial ischemia. As such, exclusion of atherosclerotic coronary ischemia by coronary angiography was considered unnecessary. Neither laboratory nor imaging findings were suggestive of myocarditis or dilated cardiomyopathy. Contrastingly, the patient’s age, previous dyslipidemia, and concomitant cyclophosphamide administration might favor the intracoronary vasospasm or thrombosis hypothesis. Since the patient outcome was favorable with optimal medical treatment, there was no need for more invasive diagnostic tests. In this case, the most likely etiology of HF is anthracycline-related myocardial damage.

## Conclusions

Cancer patients undergoing cardiotoxic treatment regimens have an increased risk of developing cardiomyopathy. The severity and reversibility of cardiac damage depend on the offending agent. Regarding anthracycline cardiotoxicity, the most important factor is the cumulative treatment dose. Early detection, the institution of cardioprotective therapies, appropriate HF treatment, and discontinuation of the drug is pivotal. The growing use of anticancer agents and the increased life expectancy of these patients justifies the need to recognize, prevent, and screen for cardiovascular toxicity.

## References

[1] Lemoniatis M (2015). Adriamycine-induced cardiomyopathy. J Med Cases.

[2] Volkova M, Russell R (2011). Anthracycline cardiotoxicity: prevalence, pathogenesis and treatment. Curr Cardiol Rev.

[3] Zamorano J, Lancellotti P, Muñoz D (2016). 2016 ESC position paper on cancer treatments and cardiovascular toxicity developed under the auspices of the ESC Committee for Practice Guidelines. Eur Heart J.

[4] Henriksen PA (2018). Anthracycline cardiotoxicity: an update on mechanisms, monitoring and prevention. Heart.

[5] Cardinale D, Iacopo F, Cipolla C (2020). Cardiotoxicity of anthracyclines. Front Cardiovasc Med.

[6] Swain AM, Whaley FS, Sewer M (2003). Congestive heart failure in patients treated with doxorubicin: a retrospective analysis of three trials. Cancer.

[7] Cruz M, Duarte‐Rodrigues J, Campelo M (2016). Cardiotoxicity in anthracycline therapy: prevention strategies [Article in Portuguese]. Rev Port Cardiol.

[8] Liu JE (2020). Anthracycline-induced cardiotoxicity: remembering the forgotten ventricle. JACC CardioOncology.

[9] Tariq H, Zahra U (2017). Doxorubicin cardiomyopathy-case report and review of histopathologic findings (RCD code: III‐1B. 5a). J Rare Cardiovasc Dis.

